# Authors’ Response to Peer Reviews of “Awareness, Experiences, and Attitudes Toward Preprints Among Medical Academics: Convergent Mixed Methods Study”

**DOI:** 10.2196/95735

**Published:** 2026-04-17

**Authors:** Mustafa Sevim, Burak Karamese, Zafer Alparslan

**Affiliations:** 1 Department of Physiology, School of Medicine, Marmara University, Başıbüyük Yolu No: 9 D:2, Istanbul, 34854, Turkey, 90 2167775500; 2 School of Medicine, Marmara University, İstanbul, Turkey

**Keywords:** preprint, medical academics, publishing attitudes, editorial policies, survey

*This is the authors’ response to peer review reports for “Awareness, Experiences, and Attitudes Toward Preprints Among Medical Academics: Convergent Mixed Methods Study.”*

## Round 1 Review

### Reviewer G [1]

*1. Abstract: The authors [2] should specify the name of the university referred to as “a major university in Istanbul.”*

*5. Methods: The authors should specify the name of the university referred to as “a major medical university in Istanbul.” This information is important for assessing the reliability of the study and for confirming ethical approval in a transparent manner.*

**Response:** The name of the university has been clearly placed where necessary.

*2. Abstract: The authors should clarify how the responding editors and the biomedical journals that were manually reviewed were selected.*

**Response:** Detailed information regarding journal policy review was covered in the “Editorial Perspectives from Turkish Biomedical Journals” and “Journal Policy Review” subsections of the “Methods” section. To better clarify this process, we added further information to related section and abstract and uploaded the whole journal list including biomedical journal categorization as a Multimedia Appendix file.

…Additionally, all responses to open-ended questions from journal editors and 118 biomedical journals were manually reviewed for their stated stance on preprints and article processing charges (APCs).

…The email was sent to the editors-in-chief of all biomedical journals, and a total of 7 editors responded…

*3. Abstract: The authors are encouraged to present concrete data rather than relying solely on descriptive summaries.*

**Response:** Additional concrete data have been added to the abstract.

...Subgroup analysis revealed that older participants scored higher on the “Preprint Test” (mean 2.20, SD 1.31 vs mean 1.97, SD 1.60) and had more experience with preprint publishing (2.5% of younger participants; 24.1% of older participants). Further, younger academics expressed less openness toward future use (17.5% in the younger group; 27.6% in the older group)...

*4. Introduction: The authors describe the study as “mixed method,” but it lacks a legitimate integration process between the qualitative and quantitative components. Therefore, the term “mixed method” should be avoided unless such integration is clearly demonstrated.*

*10. Methods: The section lacks a description of statistical analysis, making the analytical process unclear and only partially reported.*

*15. Results: The process for analyzing the qualitative responses is not clearly described, and the presentation of the results is extremely limited.*

*16. Results: The authors should provide information on how the responses from the editors were summarized. Without this explanation, reviewers and potential readers may find it difficult to interpret the results presented.*

**Response:** Following these comments, the subheading “Study Design and Data Analyses” was added to the “Methods” section of the manuscript.

Study Design and Data Analyses

This study employed a convergent mixed methods design integrating quantitative survey data, qualitative insights, and document-based content analysis to explore medical academics’ awareness and attitudes toward preprints.

A cross-sectional online questionnaire included demographic questions, Likert scales, and multiple-choice items. Descriptive statistics (frequencies, percentages, means, medians, SDs) were used and no inferential statistical testing was conducted. This survey was reported in accordance with the Checklist for Reporting Results of Internet E-Surveys (CHERRIES), and the completed checklist has been uploaded as a Multimedia Appendix file.

Quantitative comparisons of journal policies were made using frequency counts and visualized via bar plots and heatmaps, with reference to impact metrics (eg, JCI quartiles).

Open-ended responses within the survey were analyzed using pattern-based thematic analysis. Commonly expressed concerns were coded inductively to identify recurrent barriers and perceptions regarding preprint use. Responses were grouped into themes such as plagiarism concerns, lack of academic recognition, policy confusion, and ethical ambiguity.

Editorial perspectives were obtained through open-ended email queries sent to biomedical journal editors. These responses were descriptively summarized to illustrate common institutional views and infrastructure limitations regarding preprint adoption.

Findings from the three data sources were integrated during interpretation to identify convergence and divergence. Quantitative trends were contextualized with qualitative themes and policy landscape shifts, enabling a holistic understanding of both individual attitudes and institutional structures shaping preprint practices in Türkiye.

*6. Methods: The authors should indicate the total number of potential participants (ie, the total number of faculty members invited or eligible to participate).*

**Response:** The total number of medical academics, which is the targeted population, was given in the “Participant Recruitment and Data Collection” subsection of the “Methods” section.

*7. Methods: The authors should explain the rationale for dividing the age groups at 40 years.*

**Response:** The mean age of our participants was 39.56, where median was 36 and ranging through 23‐73. Moreover, age 40 can be considered as a turning point for a researcher in Türkiye. This age nearly corresponds to the time period when a researcher becomes an assistant professor. Altogether, we hypothesized that being above or below age 40 means something worth considering in terms of looking to science practice, which may affect attitude toward preprints. The necessary explanation on this subject has also been added to the “Subgroup Analyses” subsection of the “Methods” section.

Age: Participants were divided into two groups based on age; those younger than 40 and those 40 or older. This 40-year threshold was chosen for two primary reasons. First, it closely reflects the central tendency of our sample’s age distribution (mean 39.56, median 36, range 23‐73). Second, within the Turkish academic context, the age of 40 is a significant career milestone, often coinciding with the transition to an assistant professorship.

*8. Methods: The authors are encouraged to classify the biomedical journals into basic and clinical categories, in the same way that they categorized the survey respondents, even if some journals may cover both areas.*

**Response:** Categorizing the journals as either “basic science” or “clinical science” was considered at the beginning. However, upon careful evaluation, it was concluded that this distinction could not be reliably applied. Since a significant majority of journals published content covering both areas, making a clear distinction was problematic. This ambiguity was compounded by the fact that the journals themselves do not use this classification system. Therefore, to maintain methodological rigor and avoid introducing subjective bias, no categorization was implemented. For full transparency, the complete list of journals is provided in a Multimedia Appendix file, should readers wish to perform their own classification.

*9. Methods: The authors should provide a list of the journals included in this study.*

**Response:** The journal list was uploaded as a Multimedia Appendix file and necessary citations were made in the relevant lines within the text.

*11. Results: The authors should ensure that the findings are presented in alignment with the methods described. As the study does not involve a systematic review but rather a journal policy review, the current framing of the Results section may give a misleading impression.*

**Response:** As noted by the reviewer, the term “systematic review” was avoided in the relevant sections to avoid creating a misleading impression.

*12. Results: For clarity and coherence, the Results section should be reorganized to reflect the sequence of the study components—for example, starting with the questionnaire survey results, followed by the findings from the editorial and journal policy survey.*

**Response:** We thank the reviewer for this thoughtful suggestion regarding the organization of the Results section. We agree that aligning the Results with the Methods is a standard and often effective approach. Therefore, we changed the heading “Editorial Perspectives” in the Methods section to “Editorial Perspectives from Turkish Biomedical Journals.” In the “Results” section, we changed the heading “Preprint and APC Policies of Turkish Biomedical Journals” to “Journal Policy Review” and “Participant Demographics” to “Results of the Survey.”

However, the order of the “Results” section was deliberately structured thematically to create a more logical and impactful narrative for the reader. The intention here is to first establish the broader context by presenting the findings from the editorial and journal policies. We believe this landscape is essential for the reader to fully understand and interpret the significance of the individual researchers’ experiences detailed in the subsequent questionnaire survey results. This “macro-to-micro” progression strengthens the overall argument.

Therefore, after careful reconsideration of the reviewer’s point, we have respectfully retained the original order, as we are confident it best serves the clarity and coherence of our findings.

*13. Results: The authors mention the impact factor, but it is not described in the Methods section. Furthermore, the year and whether it represents the 2-year or 5-year impact factor are not specified.*

**Response:** Only the 2-year journal impact factor is used throughout this study. The 2-year journal impact factor and journal citation index (JCI) quartiles are clarified in the “Editorial Perspectives from Turkish Biomedical Journals” subsection of the “Methods” section.

From an initial list of 280 journals, 264 remained after excluding duplicates, inaccessible websites, and journals with unclear policies. The 2-year impact factors (IF) and Journal Citation Index (JCI) quartiles were obtained as well.

*14. Results: The description of the preprint test, including its content and scoring method, is insufficient, making it difficult to assess its appropriateness.*

**Response:** The meaning of “preprint test” and how this score was generated are further detailed under the subheading “Survey Instrument” in the “Methods” section. The full survey form is also included as a Multimedia Appendix file.

To quantify objective knowledge of preprints, a “preprint test score” was generated from 4 multiple-choice questions. Participants received 1 point for each correct response, resulting in a total score ranging from 0 to 4. Higher scores indicate higher knowledge of preprints. The answers given to the relevant part of the survey (9th question: “Tick the option you think is correct.”) were used to calculate the “preprint test score.” The whole survey form may be found in Multimedia Appendix 1.

*17. Discussion: The authors should revise the manuscript for logical consistency and explicitly discuss the limitations of this study prior to submitting it to another journal.*

**Response:** The manuscript has been revised and the limitations section expanded as follows:

It is important to acknowledge that this study was conducted at a single academic institution in İstanbul. As such, our findings represent a localized snapshot and should be interpreted with caution. Additionally, while the survey captured a range of perspectives, the response rate was modest, and some questions had incomplete responses. The number of editor responses was also limited, restricting the depth of qualitative analysis. Finally, the policy review was limited to publicly available information, which may not fully reflect internal editorial practices or unpublished updates. Broader, multicenter studies will be necessary to determine whether these patterns hold across other regions and institutions in Türkiye.

*18. Tables: The authors should ensure consistent use of commas, periods, and digit formatting. Furthermore, the tables contain typographical errors that need correction.*

*19. References: The authors should review the reference formatting and ensure that it adheres to the journal’s prescribed style.*

**Response:** The format of the manuscript, including references and tables, was revised and edited based on the editor’s and reviewer’s comments.

### Reviewer I [3]

*This paper offers valuable insights into attitudes toward preprinting at a medical institution in Istanbul. The research appears sound to me. The paper is clear and easy to read.*

*I have one important comment: I was unable to find the survey form. I urge the authors to make the survey form, and also the data collected in the survey, openly available. To interpret the results of the survey, it is important to have access to the underlying survey questions.*

**Response:** To ensure full interpretation of the results, the entire survey form is included as a Multimedia Appendix file.
